# Contributors to the *RSC Chemical Biology* Emerging Investigators Collection 2023

**DOI:** 10.1039/d4cb90013h

**Published:** 2024-03-19

## Abstract

This article profiles the early career researchers whose work features in the *RSC Chemical Biology* Emerging Investigators Collection 2023.
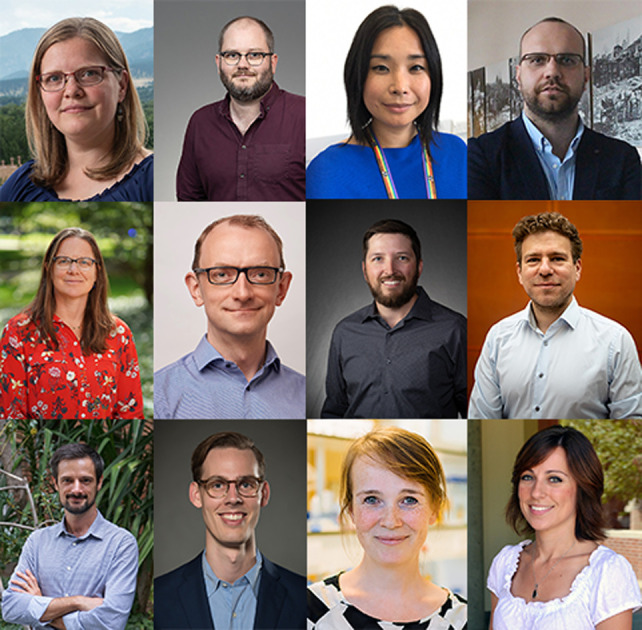


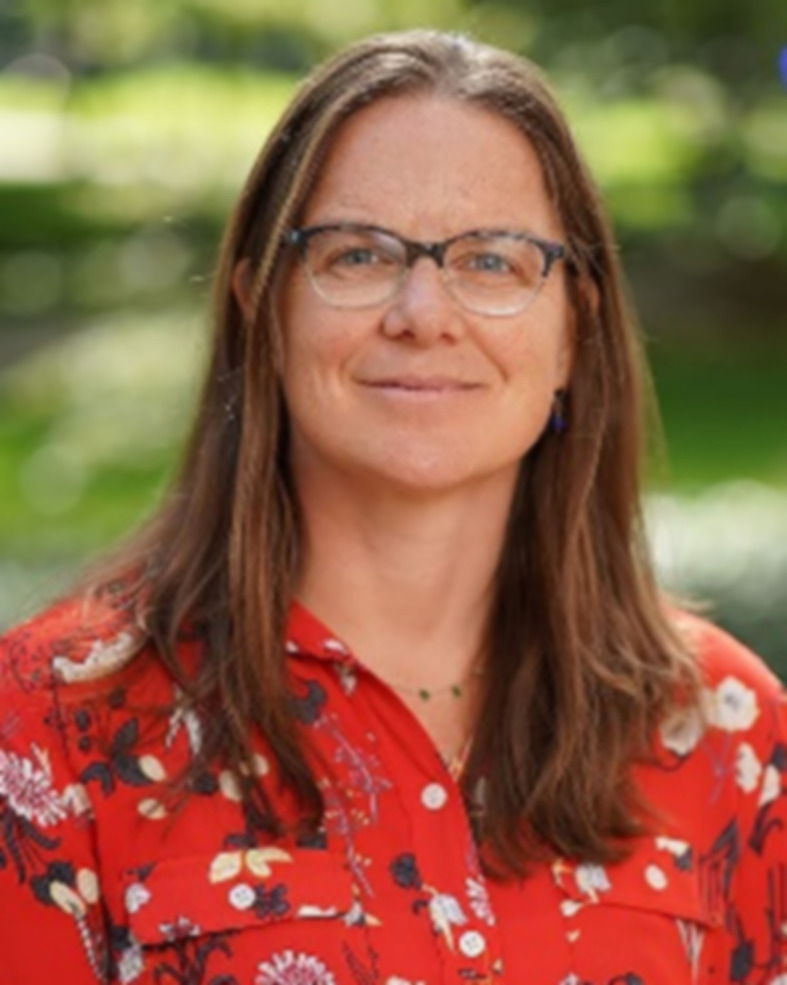
Kristin Koutmou is a biochemist working to understand how cells meet the challenge of synthesizing the correct number of proteins at the right time. She completed her PhD at the University of Michigan with Prof. Carol Fierke and post-doctoral studies in the laboratory of Prof. Rachel Green at the Johns Hopkins School of Medicine. In 2016, Dr Koutmou launched her independent research program at the University of Michigan, investigating how chemical modifications on the blueprints for protein synthesis, mRNAs, impact protein production. In 2023, she was promoted to Associate Professor and named the Dow Early Career Professor of Chemistry.

Her contribution to the 2023 *RSC Chemical Biology* Emerging Investigators collection can be read at https://doi.org/10.1039/D2CB00229A.


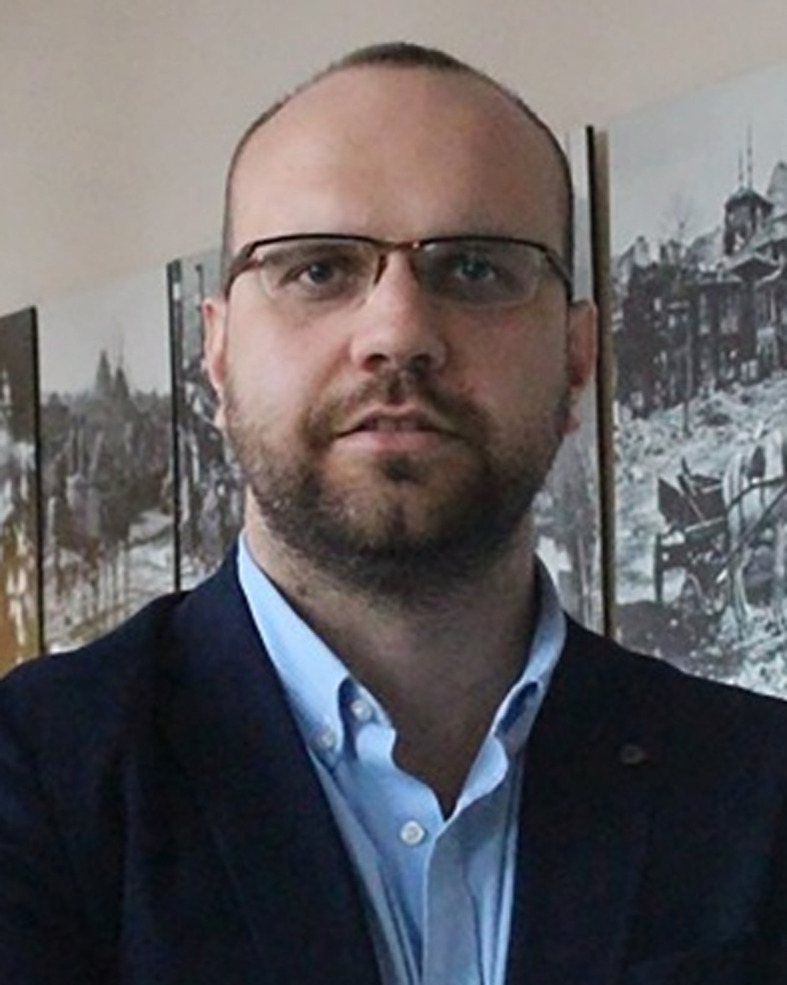
Safacan Kolemen graduated from the Chemistry Department of Bilkent University in 2008. After completing his MSc studies in the same department, he obtained his PhD from Bilkent University in 2014. Later, he worked at UC Berkeley as a postdoctoral researcher. In 2017, he joined the Department of Chemistry at Koç University as an Assistant Professor. His research focuses on the development of phototherapy and bioimaging agents. He received Outstanding Young Scientists awards from the Science Academy, Turkey, and Turkish Academy of Sciences. He is a member of the editorial advisory board of *ACS Bio & Med Chem Au* and *ACS Central Science*.

His contribution to the 2023 *RSC Chemical Biology* Emerging Investigators collection can be read at https://doi.org/10.1039/D3CB00070B.


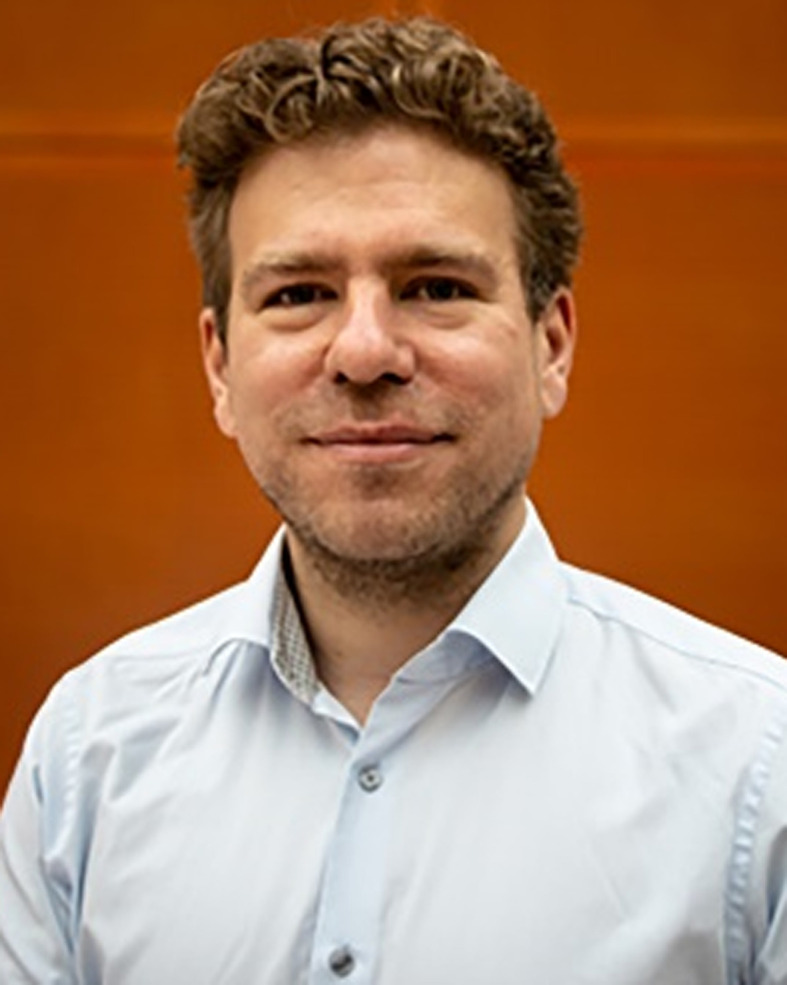
Kevin Neumann is an Assistant Professor at Radboud University in Nijmegen, the Netherlands. After obtaining his MSc in Stuttgart, Germany, he joined the group of Prof. Mark Bradley for his PhD studies at the University of Edinburgh. From 2018 to 2021, Kevin worked in the group of Prof. Jeffrey Bode as a postdoctoral fellow at ETH Zurich, Switzerland. Since 2021, Kevin has been an Assistant Professor in Nijmegen, the Netherlands, and is leading an interdisciplinary group that works in the fields of chemical biology, organic chemistry and nanomedicine. Among other things, his group focuses on the development of new click strategies for precision synthesis of therapeutic active biomolecules, including proteins and cyclic peptides.

His contribution to the 2023 *RSC Chemical Biology* Emerging Investigators collection can be read at https://doi.org/10.1039/D3CB00062A.


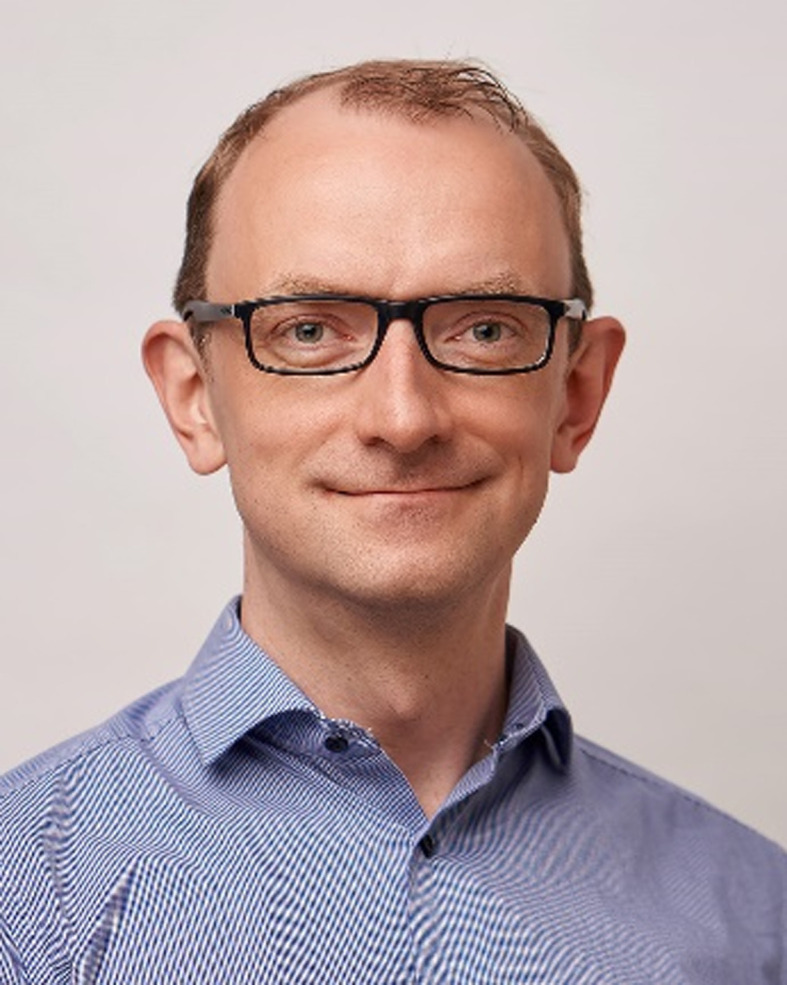
Hajo Kries is currently head of the junior research group Biosynthetic Design of Natural Products at the Leibniz Institute for Natural Product Research and Infection Biology in Jena, Germany. His research revolves around the engineering of nonribosomal peptide synthetases. After receiving his MSc and PhD in Chemistry from ETH Zurich, Switzerland, where he conducted research in the laboratory of Donald Hilvert focusing on enzyme design and engineering, he furthered his expertise as a Marie Skłodowska-Curie Fellow in the laboratory of Sarah E. O’Connor at the John Innes Center in Norwich, UK.

His contribution to the 2023 *RSC Chemical Biology* Emerging Investigators collection can be read at https://doi.org/10.1039/D3CB00061C.


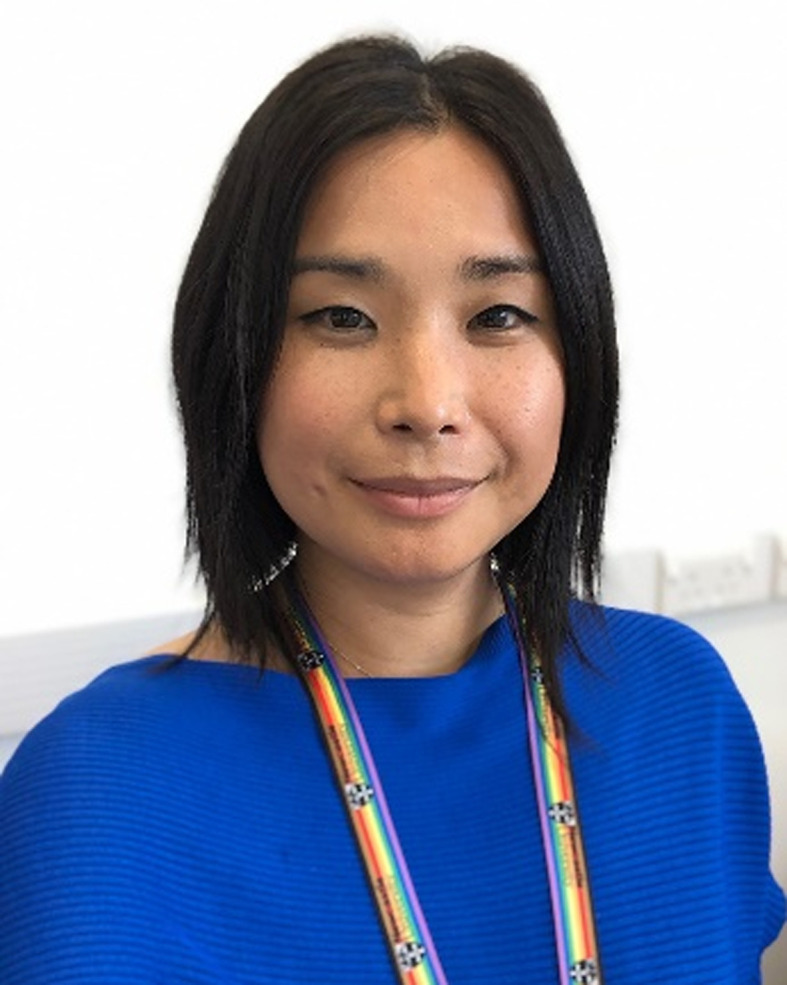
Akane Kawamura is a Chair and Professor of Chemical Biology at Newcastle University, and a visiting Professor at the University of Oxford. Research in her group centres on the development of chemical biology tools and approaches to study the biological functions of protein targets of therapeutic interest. Her recent work has focused on the application of peptide-based techniques to study and disrupt protein–protein interactions and enzyme function. In 2023, Akane received the RSC Chemistry–Biology Interface Jeremy Knowles Award, for the development and application of cutting-edge technologies and chemical probes to study and modulate therapeutically important biological processes.

Her contribution to the 2023 *RSC Chemical Biology* Emerging Investigators collection can be read at https://doi.org/10.1039/D3CB00168G.


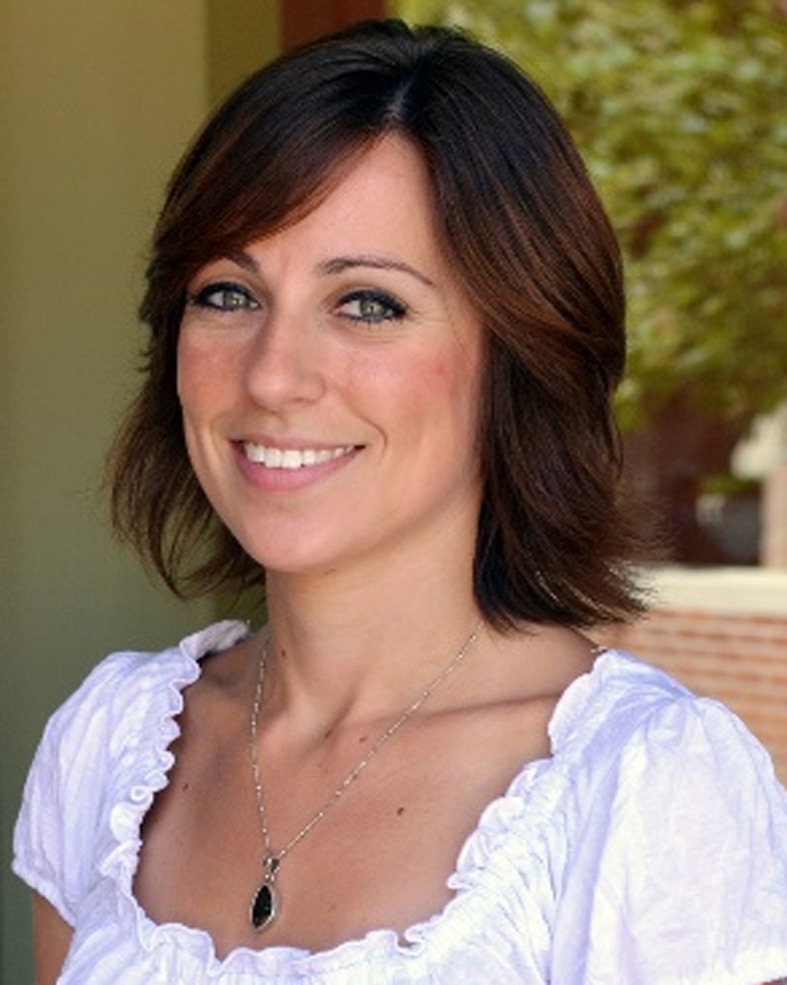
Jaclyn M. Winter is an Associate Professor at the University of Utah in the Department of Pharmacology and Toxicology. She earned her BSc in chemistry and molecular genetics from SUNY Fredonia and obtained her PhD under the guidance of Professor Bradley Moore at the Scripps Institution of Oceanography. After a short postdoc in the lab of Professor Christian Hertweck at the Hans Knöll Institute, she conducted further training with Professor Yi Tang at UCLA. Her current research program is focused on natural product biosynthesis and the bioengineering of natural product pathways from marine-derived fungi and microorganisms isolated from hypersaline environments.

Her contribution to the 2023 *RSC Chemical Biology* Emerging Investigators collection can be read at https://doi.org/10.1039/D3CB00088E.


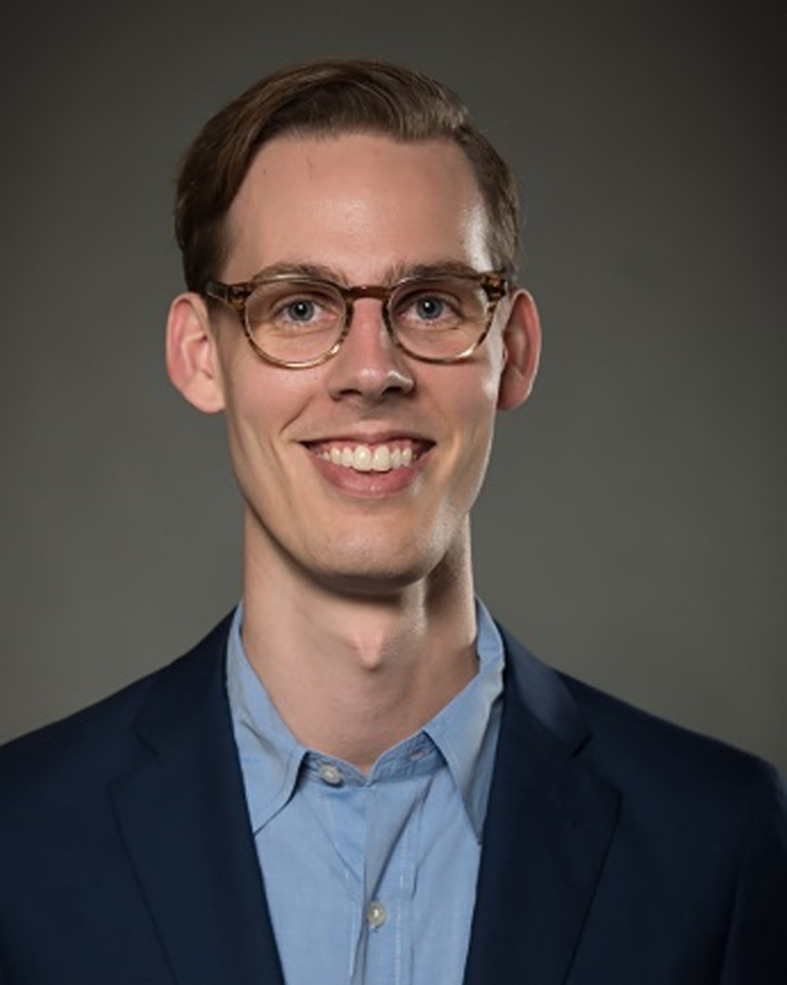
Benjamin E. Partridge is an Assistant Professor of Chemistry at the University of Rochester. After undergraduate studies at the University of Oxford, Ben moved to the USA to pursue his PhD in supramolecular chemistry with Virgil Percec at the University of Pennsylvania. Following postdoctoral work on DNA-programmed protein materials with Chad Mirkin at Northwestern University, Ben launched his independent group at the University of Rochester in 2022. His group is broadly interested in leveraging the potential of supramolecular chemistry as a synthetic discipline to design and build bioinspired noncovalent materials that can mimic, augment, and even replace natural systems.

His contribution to the 2023 *RSC Chemical Biology* Emerging Investigators collection can be read at https://doi.org/10.1039/D3CB00086A.


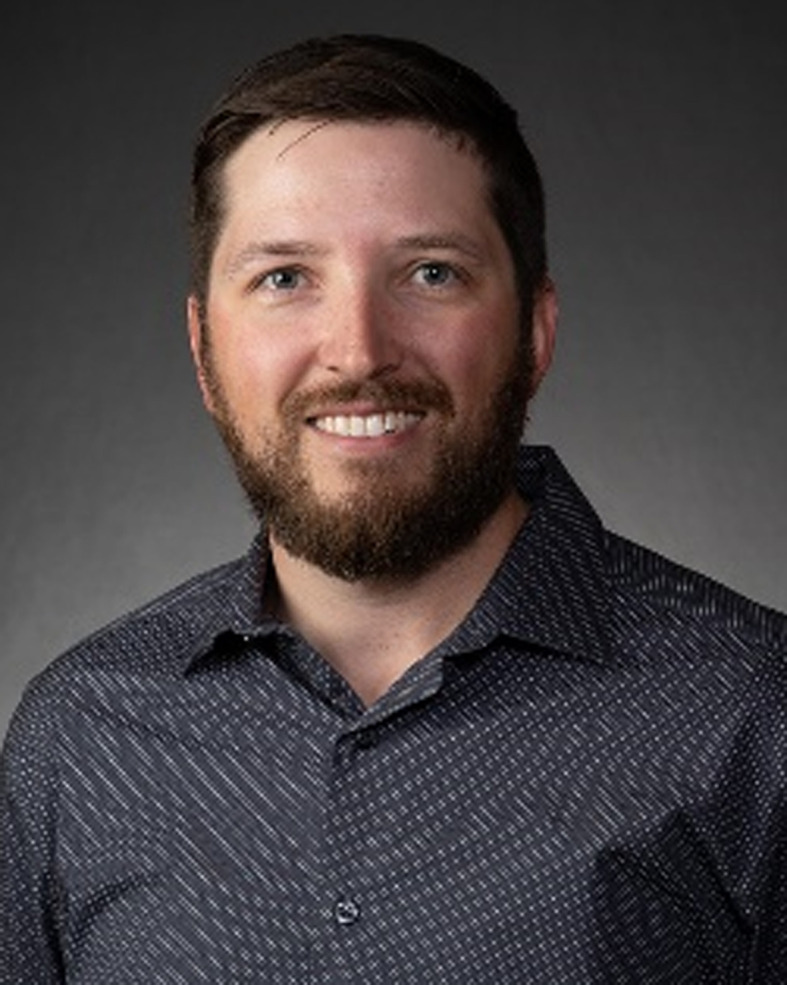
Brian Michel obtained his BS in Chemistry from Western Washington University followed by PhD studies at the University of Utah under the supervision of Matt Sigman. He then conducted postdoctoral work with Chris Chang at the University of California, Berkeley as an American Heart Association postdoctoral fellow. In 2014, he started his independent career at the University of Denver, where he is now an Associate Professor. His research interests focus on applying knowledge from catalytic systems for the detection and release of biologically relevant small molecules, such as carbon monoxide and ethylene.

His contribution to the 2023 *RSC Chemical Biology* Emerging Investigators collection can be read at https://doi.org/10.1039/D3CB00079F.


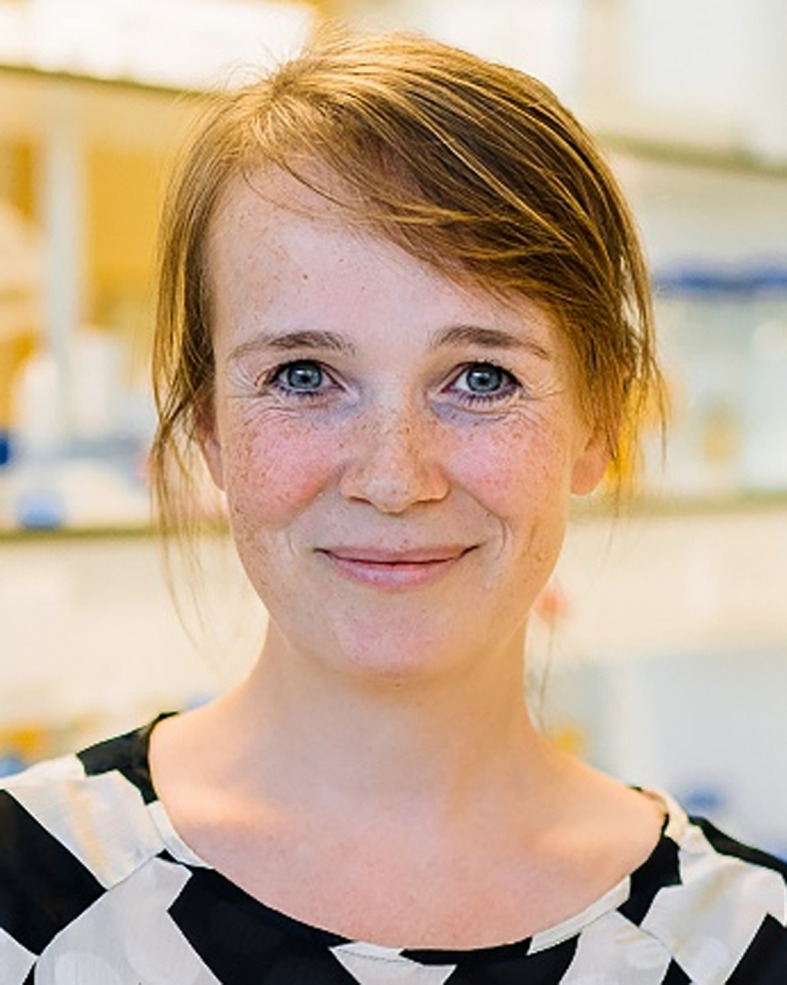
Marthe Walvoort obtained her PhD degree in 2012 (*cum laude*) at Leiden University (the Netherlands) on the organic chemistry of carbohydrates. This was followed by a postdoctoral period in the glycobiology group of Prof. Barbara Imperiali at the Massachusetts Institute of Technology (Boston, USA). At the end of 2015, Walvoort joined the University of Groningen as an Assistant Professor and Rosalind Franklin fellow in the Chemical Biology division at the Stratingh Institute for Chemistry, and she was promoted to Associate Professor in Chemical Glycobiology in 2021.

In her research, Walvoort combines her expertise in organic (carbohydrate) chemistry and biochemistry to unravel the impact of sugars in health and disease. Current research topics include bacterial adhesin glycosylation and its impact on infection, the role of lipopolysaccharides in antibiotic susceptibility, and understanding the health effects of exopolysaccharides from probiotics.

Her contribution to the 2023 *RSC Chemical Biology* Emerging Investigators collection can be read at https://doi.org/10.1039/D3CB00110E.


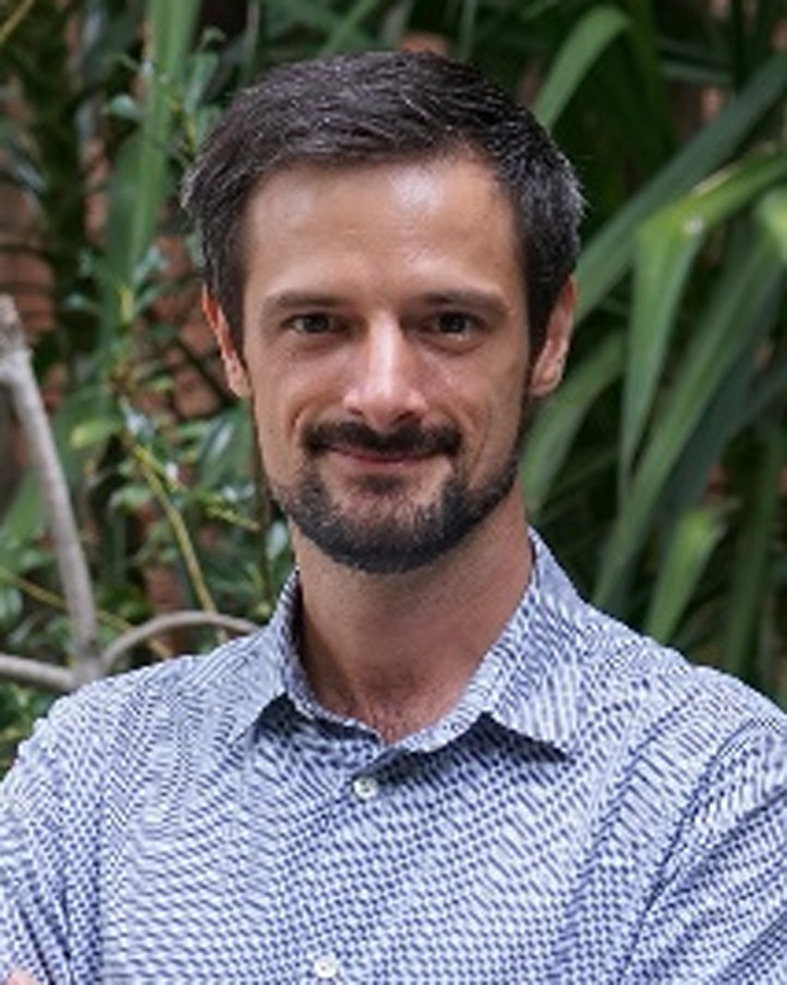
Benjamí Oller-Salvia is an associate professor at the IQS School of Engineering, Ramon Llull University. He completed his PhD in Prof. Ernest Giralt’s laboratory at IRB Barcelona and conducted postdoctoral research in Prof. Jason Chin’s group at the MRC LMB in Cambridge. With the research group he leads (ChemSynBio) and the support of the European Research Council and “la Caixa” Foundation, he combines chemical and synthetic biology to study transport across the blood–brain barrier and to generate smart biotherapeutics, especially targeting brain diseases.

His contribution to the 2023 *RSC Chemical Biology* Emerging Investigators collection can be read at https://doi.org/10.1039/D3CB00194F.


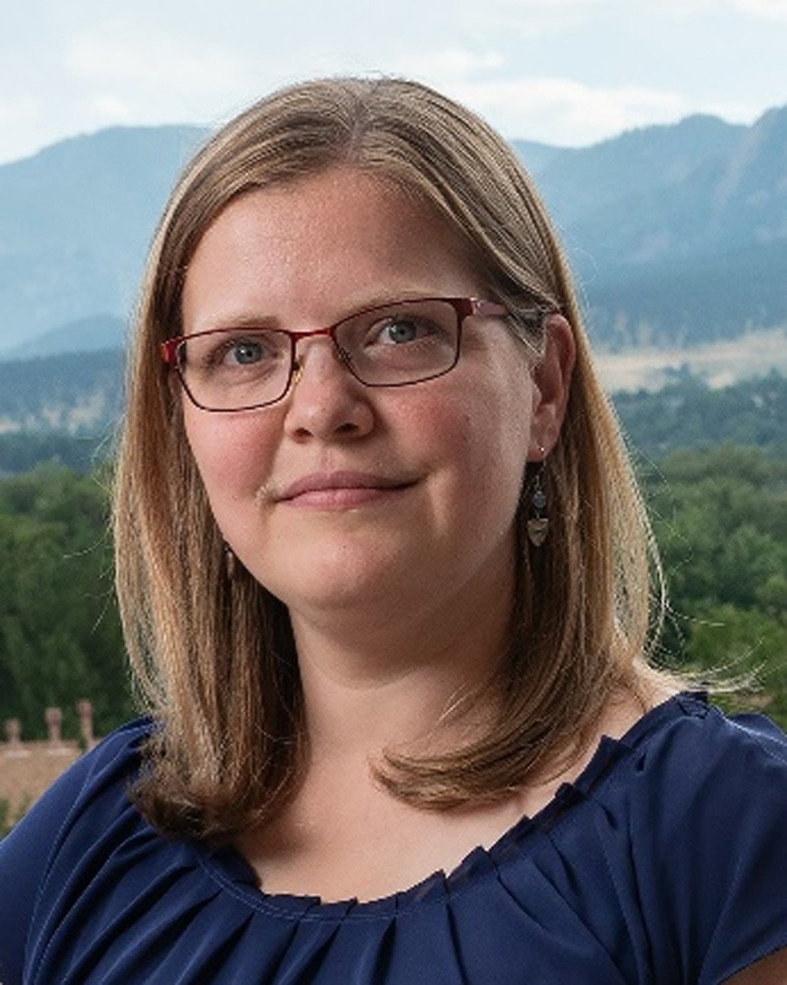
Dr Esther Braselmann is a Clare Boothe Luce Assistant Professor in the Chemistry Department at Georgetown University. Prior to this, she was a K99/R00-funded postdoctoral scholar at the University of Colorado in Boulder in the laboratory of Dr Amy Palmer where she spearheaded the development of Riboglow, a platform to visualize RNAs in live mammalian cells. The Braselmann group is exploring fluorescence lifetime imaging microscopy as an imaging modality for Riboglow. Dr Braselmann received her undergraduate degree from the University of Bielefeld, Germany, and her PhD from the University of Notre Dame with Dr Patricia Clark, working on protein folding in live cells.

Her contribution to the 2023 *RSC Chemical Biology* Emerging Investigators collection can be read at https://doi.org/10.1039/D3CB00197K.


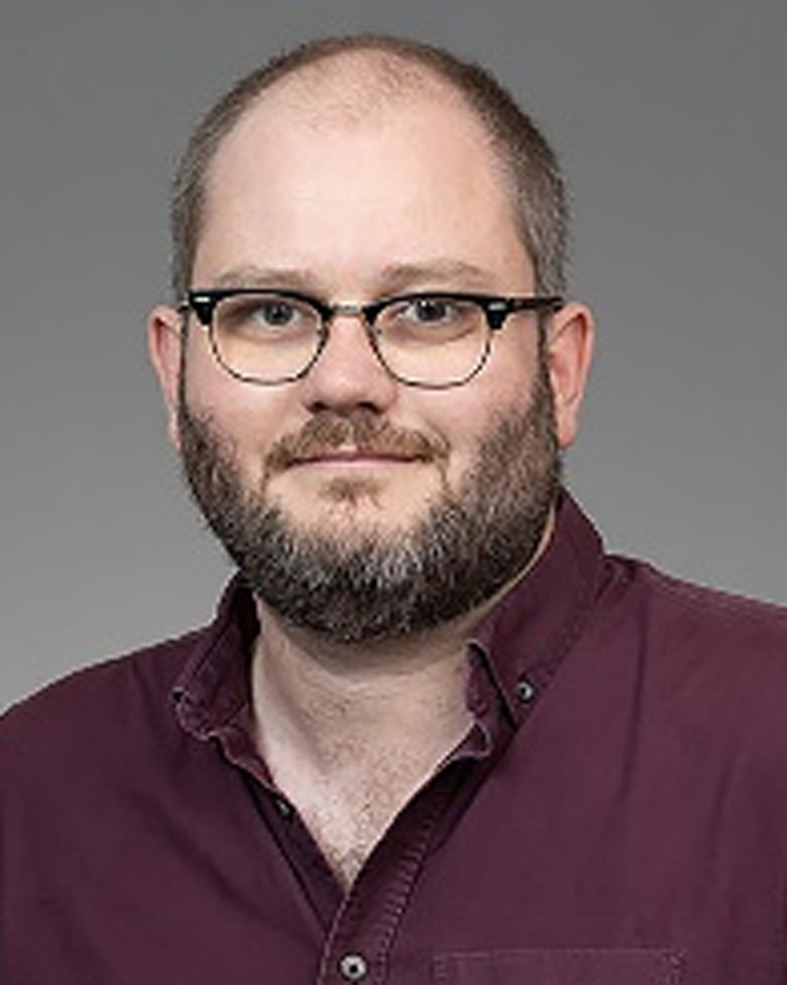
George M. Burslem obtained his undergraduate degree in Chemistry from the University of Bristol, before moving to the University of Leeds for his doctoral studies with Professors Andrew Wilson and Adam Nelson, focused on inhibiting protein–protein interactions. He was awarded his PhD in 2015 and moved to Yale University to perform postdoctoral research in the group of Professor Craig Crews. In 2020, George started his independent career at the University of Pennsylvania in the Department of Biochemistry and Biophysics, the Department of Cancer Biology, and the Penn Epigenetics Institute. The Burslem lab develops chemical tools to understand and modulate lysine post-translational modifications.

His contribution to the 2023 *RSC Chemical Biology* Emerging Investigators collection can be read at https://doi.org/10.1039/D3CB00229B.

## Supplementary Material

